# Serum Trace Elements Concentrations in Patients with Restless Legs Syndrome

**DOI:** 10.3390/antiox11020272

**Published:** 2022-01-29

**Authors:** Félix Javier Jiménez-Jiménez, Pedro Ayuso, Hortensia Alonso-Navarro, Marisol Calleja, Mónica Díez-Fairén, Ignacio Álvarez, Pau Pastor, José Francisco Plaza-Nieto, Santiago Navarro-Muñoz, Laura Turpín-Fenoll, Jorge Millán-Pascual, Marta Recio-Bermejo, Rafael García-Ruiz, Esteban García-Albea, José A. G. Agúndez, Elena García-Martín

**Affiliations:** 1 Section of Neurology, Hospital Universitario del Sureste, 28500 Arganda del Rey, Spain; hortalon@yahoo.es (H.A.-N.); marisol.calleja@salud.madrid.org (M.C.); jfrancisco.plaza@salud.madrid.org (J.F.P.-N.); 2 University Institute of Molecular Pathology Biomarkers, ARADyAL Instituto de Salud Carlos III, Universidad de Extremadura, 10071 Cáceres, Spain; payupar@unex.es (P.A.); elenag@unex.es (E.G.-M.); 3 Fundació per la Recerca Biomèdica i Social Mútua de Terrassa, 08221 Terrassa, Spain; monicadifa@gmail.com (M.D.-F.); ignacioalvafer@gmail.com (I.Á.); pastorpau@gmail.com (P.P.); 4 Movement Disorders Unit, Department of Neurology, Univeristy Hospital Mutua de Terrassa, 08221 Terrassa, Spain; 5 Section of Neurology, Hospital La Mancha-Centro, 13600 Alcázar de San Juan, Spain; satinamu@hotmail.com (S.N.-M.); lturdoc@hotmail.com (L.T.-F.); jorge.millan.pascual@gmail.com (J.M.-P.); marecber@hotmail.com (M.R.-B.); rafaelgarciaruiz@outlook.com (R.G.-R.); 6 Department of Medicine-Neurology, Hospital “Príncipe de Asturias”, Universidad de Alcalá, Alcalá de Henares, 28805 Madrid, Spain; egarciaalbea@gmail.com

**Keywords:** copper, restless legs syndrome, serum levels, trace metals, zinc

## Abstract

Increased brain and serum zinc levels in patients with idiopathic restless legs syndrome (idiopathic RLS or iRLS) were described when compared with controls, suggesting a possible role of zinc in the pathogenesis of this disease. However, serum magnesium, calcium, manganese, iron, and copper levels of RLS patients were similar to controls, suggesting a specific impairment of zinc-dependent metabolism in RLS. The aim of this study is to assess the serum concentrations of trace elements involved in oxidative stress or causing peripheral nerve toxicity in a large series of patients with iRLS and controls. We determined serum levels of iron, copper, manganese, zinc, magnesium, selenium, calcium, aluminium, lead, cadmium, arsenic and mercury in 100 patients diagnosed with iRLS and in 110 age- and sex-matched controls using Inductively Coupled Plasma Mass Spectrometry. Serum copper, magnesium, selenium, and calcium concentrations were significantly higher in RLS patients than in controls. These differences were observed both in men and women. There were no major correlations between serum trace metal concentrations and age at onset of RLS or RLS severity, nor was there any association with a family history of RLS or drug response. This study shows an association between increased serum concentrations of copper, magnesium, selenium, and calcium with RLS in a Spanish Caucasian population and does not confirm the previously reported increase in serum zinc concentrations in patients suffering from this disease, suggesting that the different accuracy of the analytical methods used could have influenced the inconsistent results found in the literature.

## 1. Introduction

Restless legs syndrome (RLS), or Willis–Ekbom disease (WED), is a sensorimotor syndrome with well-established diagnostic criteria [1] and is highly prevalent in many countries [2]. Linkage studies in families identified at least eight susceptibility loci for familial RLS [3]. Initial genome-wide association studies (GWAS) identified 6 susceptibility genes [3], confirmed in a further meta-analysis of GWAS, which identified 13 additional susceptibility loci [4]. However, these 19 loci can only explain 11.7% of RLS heritability [4]. Another recent GWAS has confirmed the 19 susceptibility loci and reported 3 novel associations [5].

The main neurochemical features found in RLS are iron deficiency and dopaminergic neuronal dysfunction. A possible contribution of GABAergic, glutamatergic, and adenosinergic neurotransmission systems to the RLS etiology has also been reported [6]. While several studies found a contribution of vitamin D deficiency [6], a recent study has described increased serum 25-hydroxyvitamin D levels in patients with iRLS compared with controls [7]. Baskol et al. [8] reported increased serum levels of malonaldehyde (MDA), which is a marker of lipid peroxidation and of advanced oxidation protein products, whereas the antioxidant thiol levels and levels of markers of nitric oxide were decreased in iRLS, suggesting a possible role of oxidative stress in the pathogenesis of iRLS.

Except for iron, a possible role of trace metals in the pathogenesis of RLS has scarcely been studied. Indeed, iron, copper, manganese, zinc, selenium, magnesium, and calcium have different biological roles, such as regulation of oxidative stress [9,10,11,12,13,14,15,16,17,18,19,20,21,22,23]. Aluminium, lead, cadmium, arsenic, and mercury damage the peripheral nervous system [11,15,24]; hence, peripheral neuropathy is a frequent cause of secondary RLS, being present even in many cases of putative iRLS [25]. Supplementary Table S1 summarizes the biological role, if any, of these trace metals and the effects of their deficiency or toxicity.

A recent study has described increased zinc levels in the substantia nigra from 4 patients with RLS compared with 4 controls, and increased serum zinc levels in 30 patients diagnosed with RLS compared with 29 controls, suggesting a role of zinc in the pathogenesis of RLS [26]. In the same study, serum levels of magnesium, calcium, manganese, iron, and copper did not differ significantly between RLS patients and controls. [26]. Another previous study showed similar cerebrospinal fluid and serum magnesium levels in 11 RLS patients and 9 controls [27].

The main objective of the present study was to replicate the previously reported increase in serum zinc concentrations [26] in a larger series of Spanish Caucasian patients diagnosed with iRLS and in healthy controls. We also assessed in these study groups the serum levels of several trace elements involved in oxidative stress or causing peripheral nerve toxicity, with the aim of establishing a possible association with RLS development. As a secondary analysis, we studied the possible relationship of serum trace element levels with several variables, including age at onset and RLS severity, gender, positive family history of RLS, and response to dopaminergic, GABAergic drugs, and to clonazepam.

## 2. Patients and Methods

### 2.1. Patients and Controls

We studied the serum concentrations of iron, copper, manganese, zinc, magnesium, selenium, calcium, aluminium, lead, cadmium, arsenic, and mercury in 100 patients diagnosed with iRLS according to the International Restless Legs Syndrome Study Group (IRLSSG) diagnostic criteria after exclusion of secondary causes of RLS, as described in detail elsewhere [28], and 110 age- and gender-matched controls.

Recruitment of iRLS patients was done in the Movement Disorders Units of the participant hospitals on the patients’ first visit. Diagnosis of RLS was made by asking in person the IRLSSG diagnostic criteria [1], including the absence of RLS-mimics. To exclude secondary causes of RLS, all patients underwent laboratory studies (blood count, routine biochemistry, serum levels of thyroid hormones, folic acid, vitamin B12, and iron metabolism studies, proteinogram, immunological studies including antinuclear antibodies, rheumatoid factor, and nerve conduction studies). Specific diagnosis with iRLS required the absence of other previous neurological diseases, and the exclusion of possible causes of secondary RLS such as peripheral neuropathy, renal failure, iron-deficiency, anaemia, rheumatoid arthritis, and exposure to drugs able to induce or to aggravate RLS, such as neuroleptics or other dopamine receptor blocking agents, antidepressants, antihistamines, or other drugs. Controls, most of them recruited from the University of Extremadura (staff or students), were healthy Caucasian Spanish individuals who did not report either a personal or familial history of RLS, tremor, or other movement disorders. Demographic data from both iRLS patients and control groups are summarized in Table 1.

In addition to the previously described exclusion criteria applied for diagnosis with iRLS, both iRLS patients and controls involved in this study were not under therapy with vitamins, calcium or mineral supplements (including iron), steroids, diuretics, diphosphonates, or drugs that could affect mineral metabolism. Participants did not have obesity or undernutrition; they did not suffer from kidney, liver, thyroid, parathyroid, oncologic or acute infectious diseases; they had no atypical dietary habits (i.e., diets consisting exclusively of one type of foodstuff such as vegetables, and others); they had not received blood transfusions; and they had no recent history of traumatism or surgery. Pregnant women were also excluded.

None of the iRLS patients were under specific therapy for this disorder at the time of enrolment in the study. Treatment with dopaminergic drugs, GABAergic drugs, and/or clonazepam was initiated after blood extraction, and the response to these therapies was assessed in the follow-up visits at 3, 6, 9, and 12 months after starting the treatment. A total of 90 patients were treated with dopamine agonists (86 of them reported total or partial improvement), 26 with GABAergic drugs (22 with total or partial improvement), and 41 with clonazepam (39 with total or partial improvement).

### 2.2. Ethical Aspects

The Ethics Committees of the Hospital La Mancha-Centro (Alcázar de San Juan, Ciudad Real, Spain), University Hospital “Príncipe de Asturias” (Alcalá de Henares, Madrid, Spain), University Hospital “Infanta Cristina” (Badajoz, Spain), and the Investigation Committee of the University Hospital of Sureste (Arganda del Rey, Madrid, Spain) approved the study protocols. The study fulfilled the principles of the Declaration of Helsinki, and written, informed consent was obligatory for inclusion.

### 2.3. Determinations of Serum Trace Metals Levels

Venous blood samples were obtained from each fasted patient or control between 8.00 and 10.00 a.m. The blood samples were immediately centrifuged, and the serum specimens obtained were frozen at −30 °C and protected from light exposure until analysis. The determinations were performed blindly using an Inductively Coupled Plasma Mass Spectrometer model 7900 (Agilent Technologies, Santa Clara, CA, USA). A total of 150 μL of serum were diluted with 4850 μL of 1% nitric acid and then injected into the spectrometer. Rhodium (400 μg/L) was used as an internal standard. The instrument parameters are shown in Table 2. All determinations were performed in triplicate.

### 2.4. Statistical Analysis

Statistical analysis was performed with the SPSS 19.0 version for Windows (SPSS Inc., Chicago, IL, USA). Comparison of serum levels of trace metals between idiopathic RLS patients and controls was made by means of a *t*-test for independent samples (for those values that followed a normal distribution according to a Kolmogorov–Smirnov test) or with the Mann–Whitney test (for those that did not follow a normal distribution). False Discovery Rate (FDR) correction was applied for adjustments for multiple analyses. Correlations of the different concentrations of serum trace metals with age at onset of idiopathic RLS and with the International Restless Legs Syndrome Study Group Rating Scale (IRLSSGRS) [29], used to assess the severity of RLS at the time of enrolment (previous to RLS treatment), were also calculated by linear regression analysis. Multivariate analyses were carried out, including variables with *p* values equal to or below 0.1.

As a secondary analysis, we calculated the differences in levels of serum trace metals in RLS patients depending on their family history of RLS and their response to dopaminergic or GABAergic drugs and clonazepam. STROBE checklist is summarized in Supplementary Table S2.

## 3. Results

Included in the study were 100 iRLS patients and 110 controls, and of these we were able to determine serum levels of 99 patients and 109 controls. Some of the elements analyzed were below the detection limits for most participants. Table 3 shows the main findings obtained, the normality tests, and the intergroup comparison values.

No patients or control subjects had cadmium levels above the detection limit, and only four patients and no control individuals had detectable levels of aluminium. For these two elements, aluminium and cadmium, intergroup comparison values could not be obtained. This aside, detectable levels of arsenic were observed in 14 patients and 28 control subjects, lead levels above the detection limit were observed in 2 patients and 8 control subjects, manganese levels above the detection limit were observed in 9 patients and 9 controls, and mercury levels were detectable in 8 patients and 4 controls. The rest of the elements were detected in 99 patients and 109 control subjects.

After performing a normality test, arsenic, iron, manganese, and zinc values did not follow a normal distribution and therefore the levels for these elements were compared using the Mann–Whitney test. For the rest of the elements, the comparisons shown in Table 3 correspond to t-tests for independent samples. Increased concentrations of calcium, copper, magnesium, and selenium were observed in RLS patients compared to control subjects. For the remaining elements, zinc, arsenic, iron, lead, manganese, and mercury, no significant differences were observed between RLS patients and controls.

Having found increased calcium levels in RLS patients (Table 3), and since we had previously described increased levels of 25-hydroxyvitamin D in RLS patients [7], we analyzed the putative correlation between calcium and 25-hydroxyvitamin D levels in the RLS patients included in this study. Supplementary Figure S1 shows the correlation plot. Linear regression analysis indicates a lack of association between these values (*p* = 0.870).

In addition, we analyzed the putative correlations between serum levels of the elements determined in this study, and both age at onset and IRLSSGRS. Results are summarized in Table 4.

Linear regression analyses only revealed weak associations for calcium and magnesium with age at onset, and for manganese with the IRLSSGRS score. The correlation plots are shown in Supplementary Figures S1–S4. In all, associations are weak and probably due to chance because of the high number of comparisons carried out and because of the small number of patients with manganese values over the detection threshold. We analyzed the putative association of serum concentrations and sex, family history of RLS, and drug response. Table 5 shows that the major associations identified in the whole study group, that is, increased concentration of calcium, copper, magnesium, and selenium among RLS patients, were consistently replicated in the analyses stratified by sex.

We also identified a weak association of increased arsenic concentrations in males with RLS compared to controls. No association whatsoever was identified with family history of RLS (Table 6), and very weak associations were observed when stratifying patients by drug response (Table 7).

## 4. Discussion

In the current study, we could not confirm the results of the previous report that found increased serum zinc concentrations in patients diagnosed with RLS [26]. Moreover, we observed a statistically non-significant decreased average concentration of zinc in RLS patients compared to control individuals. In any case, comparisons of zinc levels suggest that the differences (if any) were in the opposite direction to those described in the previous study with a smaller sample size [26]. Interestingly, a recent study has described significantly lower serum levels of zinc in 119 pregnant women with RLS compared with 134 “healthy” pregnant women (none of the women included in our study were pregnant) [30]. These results suggest that, at least to date, serum zinc concentrations cannot be considered as a reliable marker for iRLS, although they do not preclude the possibility that zinc could be increased in certain brain areas, as previously described [26].

The results of the current study have shown raised serum concentrations of copper, magnesium, selenium, and calcium in RLS patients compared to control subjects, the statistical significance in all cases being very high. Although the results of our study show a weak association of increased arsenic concentrations in males with RLS compared to controls, this is based only on two patients, and therefore is likely due to chance. The very weak associations observed when stratifying patients by drug response (Table 7) are probably due to chance as well, because of the limited size of the groups. Previous studies with a considerably small sample size have described similar serum levels of copper [26], magnesium [26,31], and calcium [26], and similar magnesium cerebrospinal fluid levels [31], in patients with iRLS compared with controls. To our knowledge, there are no previous reports on serum selenium levels in RLS patients.

Magnesium deficiency has been considered to be possibly related to the presence of periodic limb movements during sleep (PLMS) and iRLS in the general population [31,32] and in patients with end-stage renal disease (ESRD) under haemodialysis [33] by some studies. Moreover, improvement of iRLS and PLMS by magnesium supplementation has been reported [34,35], although a systematic review did not find a significant effect of magnesium in this condition [36]. Two studies found similar serum magnesium levels in pregnant patients diagnosed with RLS compared with pregnant patients without RLS [37] and in patients with ESRD with RLS compared with patients with ESRD without RLS [38]. However, a recent study has described significantly lower serum levels of magnesium in pregnant women with RLS compared with 134 “healthy” pregnant women [30]. In contrast, a prospective case-control questionnaire study for RLS involving 600 pregnant women showed a significantly higher magnesium intake in patients with RLS than in those without RLS [39]. In addition, a recent retrospective study involving 578 ESRD patients receiving maintenance haemodialysis for more than 1 year has shown an association of pre-dialysis hypermagnesemia both with uraemic RLS and with increased morbidity of this syndrome [40]. The results of these studies [39,40] are along the same lines as our finding of increased serum magnesium levels in patients with iRLS (none of the women included in our study were pregnant) in comparison with controls.

Information regarding the possible role of selenium in RLS is limited to an anecdotal report suggesting the presence of RLS associated with other “muscular symptoms” in patients with selenium deficiency [41]. Because of their safety, and antioxidant and dopaminergic promoting action, selenium was used as possible therapy for RLS in a clinical trial at doses of 50 and 200 μg and compared with placebo in a cross-over study, showing a significant short-term improvement in RLS symptoms in patients treated with the two doses of selenium [42]. Improvement with selenium of three patients with RLS was reported in a further study [43].

Serum calcium levels have been found to be similar in patients with ESRD under haemodialysis with RLS to those without RLS in several studies [37,38,41,42,43,44,45,46,47] and in a meta-analysis [48]. However, other studies have found increased serum calcium levels in patients with RLS [49] and an association of higher serum calcium levels with the severity of RLS associated with ESRD [50]. Calcium urinary excretion in patients with iRLS was similar during day and night-time [51]. Serum calcium levels have been found to be similar in patients with diabetes mellitus type 2 with RLS when compared with those without RLS [52], and in pregnant women with RLS compared with those without RLS [30]. Finally, a prospective case-control questionnaire study for RLS syndrome involving 600 pregnant women showed a significantly higher calcium intake in patients with RLS than in those without RLS [39]. The results of several of these studies [39,49,50] are along the same lines as our finding of increased serum calcium levels in patients with iRLS in comparison with controls. To our knowledge, there are no data regarding serum copper levels in RLS associated with other pathologies. Even though we previously reported increased serum 25-hydroxyvitamin levels in the same series [7], these values and serum calcium levels were not correlated in our patients (Supplementary Figure S2).

Although abundant data suggest a crucial role of brain iron deficiency in the pathogenesis of iRLS, and the fact that iron deficiency is a well-known cause of secondary RLS [6], three previous studies [53,54,55], one of them with a large size sample [55], have shown, as in the present study, normality of serum/plasma iron levels in patients diagnosed with iRLS, whereas only one study with a very small sample size found decreased iron levels [56].

These results are indeed difficult to interpret. Although we did not study total antioxidant capacity and/or serum level of reactive oxygen species, a previous study found increased serum malondialdehyde and plasma advanced oxidation protein product levels, and decreased serum thiol and nitric oxide levels in patients with RLS compared with controls, suggesting a possible role of oxidative stress in the pathogenesis of this disease [8]. For this reason, we speculate that increased serum copper levels may be related to the presence of oxidative stress, while increased serum magnesium and selenium levels could be related to an attempt to protect against oxidative stress as well. In addition, copper can impair iron homeostasis [10,11], iron deficiency being one of the main neurochemical features of RLS [6].

The main limitations of the current study are the facts that the female-to-male ratio is relatively high and that recruitment of RLS patients was carried out in the Movement Disorders Units. The first is a frequent limitation of studies on RLS because the prevalence of RLS is higher in women in both epidemiological and in clinical studies. However, RLS and control groups were sex-matched, and the results obtained by stratifying by gender were similar. The second limitation could be selection bias, since it is more likely that RLS patients from these Units were those with the most severe symptoms. In contrast, in the present study, the statistical power for serum trace elements was high. Taking into account these limitations, our results could indicate a possible association between increased serum copper, magnesium, selenium, and calcium levels and RLS (although these data need to be replicated) and do not confirm the previously reported increase in serum zinc levels [26].

## Figures and Tables

**Table 1:** Demographic and clinical data of the series studied.

Group	RLS Patients (*n* = 100)	Healthy Controls (*n* = 110)	*p* Values
Age (years): mean (SD); range	59.16 (13.52); 22–94	60.24 (5.23); 50–68	0.895
Age at onset (years): mean (SD); range	42.87 (17.76); 5–82	NA	
Female N (%)	70 (70.0)	75 (68.2)	0.776
Positive family history: N (%)	80 (80.0)	NA	
IRLSSG scale score, mean (SD); range	24.65 (6.19); 6–40	NA	

**Table 2:** Instrumental conditions for the analysis.

Parameter	Value
Potency RF (W)	1550
Plasma Mode	General Purpose
Omega Bias (V)	−120
Omega lens (V)	9.3
Extract 2 (V)	−245
Deflect Lens (V)	1.0
Energy discrimination (V)	5
Collision gas (mL/min)	5
Cell Entrance (V)	−40
Cell Exit (V)	−60

**Table 3:** Summary of the results obtained.

Element	Detection Limit	Patients over the Detection Limit: No. (Mean; SD, Min-Max)	Controls over the Detection Limit: No. (Mean; SD, Min-Max)	Normality Test (Kolmogorov-Smirnov)	Intergroup Comparison Values. Mean Difference (95% CI); *p* (*t*-Test or MW-Test)
Aluminium	150 μg/L	4 (215.45; 50.42, 179.80–251.1)	0 (--)	--	--
Arsenic	5 μg/L	14 (32.35; 55.01, 5.07–212.9)	28 (10.91; 9.03, 5.07–52.3)	*p* < 0.001	21.44 (0.12 to 42.76); 0.107
Cadmium	5 μg/L	0	0	--	--
Calcium	5 mg/L	99 (133.23; 22.08, 78.26–179.10)	109 (108.65; 18.97, 52.93–162.60)	*p* = 0.329	24.59 (18.97 to 30.20); <0.0001
Copper	30 μg/L	99 (1540.25; 400.20, 733.44–2240.20)	109 (1181.11; 337.31, 595.27–2309.60)	*p* = 0.169	306.49 (199.45 to 413.52); <0.0001
Iron	30 μg/L	99 (1553.51; 491.44, 818.46–4354.20)	109 (1654.38; 1024.05, 586.73–8612.50)	*p* < 0.001	−100.87 (−324.03 to 122.29); 0.296
Lead	5 μg/L	2 (8.95; 4.31, 5.91–12.00)	8 (13.44; 7.97, 5.73–30.70)	*p* = 0.586	−4.49 (−18.35 to 9.38); 0.477
Magnesium	5 mg/L	99 (29.67; 4.72, 14.43–38.80)	109 (23.68; 4.18, 11.21–35.70)	*p* = 0.599	5.22 (3.94 to 6.51); <0.0001
Manganese	5 μg/L	9 (8.06; 3.60, 5.10–14.90)	9 (5.84; 0.57, 5.13–6.90)	*p* = 0.001	2.20 (−0.38 to 4.78); 0.601
Mercury	5 μg/L	8 (13.04; 14.73, 5.17–15.50)	4 (9.83; 5.56, 5.61–18.00)	*p* = 0.152	3.21 (−14.11 to 20.53); 0.688
Selenium	5 μg/L	99 (122.93; 26.31, 58.80–188.9)	109 (102.14; 24.42, 44.62–195.10)	*p* = 0.927	20.79 (13.85 to 27.72); <0.0001
Zinc	30 μg/L	99 (1164.31; 195.91, 814.47–1756.90)	109 (1569.09, 802.51, 624.47–3577.70)	*p* < 0.001	−404.77 (−568.07 to −241.48); 0.181

**Table 4:** Correlation between serum levels, age at onset and IRLSSGRS.

Element	Age at Onset (*p* Value; Standardized Beta Coefficient)	IRLSSGRS (*p* Value, Standardized Beta Coefficient)
Arsenic	*p* = 0.912; Beta coefficient = 0.033	*p* = 0.139; Beta coefficient = −0.416
Calcium	*p* = 0.051; Beta coefficient = 0.196	*p* = 0.569; Beta coefficient = 0.058
Copper	*p* = 0.192; Beta coefficient = 0.132	*p* = 0.536; Beta coefficient = 0.063
Iron	*p* = 0.337; Beta coefficient = −0.090	*p* = 0.893; Beta coefficient = 0.014
Lead	*p* = 0.213; Beta coefficient = −0.495	*p* = 0.436; Beta coefficient = −0.323
Magnesium	*p* = 0.091; Beta coefficient = 0.171	*p* = 0.787; Beta coefficient = −0.028
Manganese	*p* = 0.429; Beta coefficient = −0.303	*p* = 0.046; Beta coefficient = 0.676
Selenium	*p* = 0.997; Beta coefficient = 0.000	*p* = 0.561; Beta coefficient = −0.059
Zinc	*p* = 0.523; Beta coefficient = 0.065	*p* = 0.869; Beta coefficient = 0.017

**Table 5:** Summary of the results obtained stratified by gender.

Element	Patients (Men) No. (Mean; SD, Min-Max)	Controls (Men): No. (Mean; SD, Min-Max)	Intergroup Comparison Values. Mean Difference (95% CI); *p* (*t*-Test or MW-Test)	Patients (Women) No. (Mean; SD, Min-Max)	Controls (Women): No. (Mean; SD, Min-Max)	Intergroup Comparison Values. Mean Difference (95% CI); *p* (*t*-Test or MW-Test)
Arsenic	2 (119.41; 132.22, 25.92 to 212.90)	9 (14.49; 14.63, 5.07 to 52.35)	12.77 (−12.90 to 38.44); 0.017	12 (17.82; 19.48, 5.07 to 69.30)	19 (9.19; 4.21, 5.11 to 20.16)	25.17 (−4.53 to 54.89); 0.070
Calcium	29 (133.85; 27.08, 78.26–159.30)	34 (107.20; 17.78, 78.20 to 154.74)	26.64 (15.25 to 38.03); <0.0001	70 (132.98; 19.86, 103.88 to 179.11)	75 (109.30; 19.56, 52.93 to 162.74)	23.68 (17.20 to 30.15); <0.0001
Copper	29 (1523.70; 407.76, 733.44–2255.59)	34 (1045.83; 273.75, 628.23 to 1797.20)	477.86 (305.07 to 650.65); <0.0001	70 (1547.10; 399.80, 976.07 to 2240.23)	75 (1242.34; 347.01, 595.27 to 2309.59)	304.67 (181.99 to 427.34); <0.0001
Iron	29 (1501.39; 423.36, 821.20–2527.77)	34 (1963.16; 1370.50, 792.78 to 8612.46)	−461.78 (−991.49 to 67.94); 0.928	70 (1575.10; 518.33, 818.46 to 4354.18)	75 (1514.40; 793.27, 586.73 to 4909.90)	60.70 (−160.96 to 282.36); 0.285
Lead	0 (-,-,-)	4 (18.69; 8.45, 11.92 to 30.70)	--	2 (8.95; 4.30, 5.91 to 11.99)	4 (18.20; 1.84, 5.73–9.60)	0.75 (−5.68 to 7.19); 0.762
Magnesium	29 (28.27; 5.39, 14.43–38.22)	34 (23.34; 4.08, 18.22–35.66)	5.03 (2.63–7.41); <0.0001	70 (29.11; 5.11, 22.66–38.75)	75 (23.82; 4.24, 11.21–34.71)	5.28 (3.74–6.82); <0.0001
Manganese	5 (7.97; 41.18, 5.10 to 14.89)	6 (6.12; 0.53, 5.79–6.92)	1.84 (−3.20 to 6.88); 0.529	4 (8.17; 3.35, 5.15 to 12.01)	5 (5.61; 0.53, 5.13–6.42)	256 (−0.98 to 6.09); 0.298
Mercury	2 (12.80; 3.80, 10.11 to 15.49)	3 (10.75; 6.58, 5.61–18.03)	2.22 (−14.63 to 19.07); 0.703	6 (13.11; 17.36, 5.17 to 48.54)	1 (7.62; -, -)	5.48 (−26.2 to 37.16); 0.687
Selenium	29 (125.69; 30.68, 58.80–212.90)	34 (102.26; 22.74, 59.48–148.37)	23.43 (9.95 to 36.92); 0.0009	70 (121.79; 24.43, 52.44 to 184.30)	75 (102.09; 25.29, 44.62 to 195.14)	19.69 (11.52 to 27.87); <0.0001
Zinc	29 (1144.55; 199.27, 814.47–1561.04)	34 (1568.43, 840.84, 640.53–3577.70)	−423.88 (−743.84 to −101.93); 0.645	70 (1172.50; 195.37, 603.60 to 1756.88)	75 (1569.39; 790.34, 624.47 to 3289.57)	−396.87 (−588.90 to −204.87); 0.347

**Table 6:** Summary of the results obtained stratified by family history or RLS.

Element	Positive Family History: No. (Mean; SD, Min-Max)	Negative Family History: No. (Mean; SD, Min-Max)	Intergroup Comparison Values. Mean Difference (95% CI); *p* (*t*-Test or MW-Test)
Aluminium	1 (599.64; -, -)	3 (210.40; 36.71, 179.80 to 251.10)	--
Arsenic	12 (36.06; 58.91, 50.7 to 212.90)	2 (9.99; 3.30, 7.65 to 12.32)	26.08 (−67.79 to 119.94); 0.556
Calcium	78 (134.02; 22.14, 72.87 to 222.70)	18 (128.53; 22.92, 78.26 to 162.07)	5.49 (−6.08 to 17.07); 0.348
Copper	78 (1548.17; 411.36, 789.05 to 2884.45)	18 (1430.42; 324.14, 733.44 to 1906.28)	117.75 (−88.37 to 323.87); 0.260
Iron	78 (1553.40; 519.97, 555.94 to 4354.18)	18 (1541.34; 361.95, 821.20 to 2233.37)	12.05 (−245.02 to 269.13); 0.645
Lead	2 (8.95; 4.31, 5.91–12.00)	0	--
Magnesium	78 (28.94; 5.05, 15.72 to 41.75)	18 (28.53; 6.12, 14.43 to 36.94)	0.41 (−2.33 to 3.14); 0.769
Manganese	5 (7.53; 3.24, 5.02 to 12.01)	3 (8.66; 5.41, 5.15 to 14.89)	−1.13 (−8.45 to 6.18); 0.717
Mercury	7 (12.68; 15.90, 5.17 to 48.54)	1 (15.49; -, -)	--
Selenium	78 (122.31; 24.72, 52.44 to 184.30)	18 (122.30; 33.20, 58.80 to 188.95)	0.01 (−13.73 to 13.74); 0.999
Zinc	78 (1168.47; 195.84, 603.60 to 1756.88)	18 (1142.79; 211.84, 685.64 to 1532.52)	25.68 (−77.55 to 128.91); 0.645

**Table 7:** Association between trace metals and medication response.

Element	Positive Response to Dopaminergic Drugs No. (Mean; SD, Min-Max)	Negative Response to Dopaminergic Drugs No. (Mean; SD, Min-Max)	Intergroup Comparison Values. Mean Difference (95% CI); *p* (*t*-Test or MW-Test)	Positive Response to Gabaergic Drugs No. (Mean; SD, Min-Max)	Negative Response to Gabaergic Drugs No. (Mean; SD, Min-Max)	Intergroup Comparison Values. Mean Difference (95% CI); *p* (*t*-Test or MW-Test)	Positive Response to Clonazepam No. (Mean; SD, Min-Max)	Negative Response to Clonazepam No. (Mean; SD, Min-Max)	Intergroup Comparison Values. Mean Difference (95% CI); *p* (*t*-Test or MW-Test)
Aluminium	4 (307.71; 196.91, 179.80 to 599.64)	0	--	1 (200.30; -, -)	0	--	3 (350.35; 217.38, 200.30–599.64)	0	--
Arsenic	11 (35.48; 61.59, 5.36 to 212.90)	1 (44.98; -, -)	--	5 (17.83; 16.06, 5.36 to 44.98)	1 (8.42; -, -)	--	7 (45.49; 75.14, 5.07 to 212.90)	0	--
Calcium	86 (133.61; 22.59, 72.87 to 222.70)	4 (137.75; 21.27, 111.37 to 156.09)	−4.13 (−27.06 to 18.78); 0.721	22 (131.57; 18.68, 81.20 to 162.07)	4 (147.55; 15.65, 129.68 to 167.54)	−15.98 (−36.55 to 4.58); 0.122	39 (132.37; 24.53, 78.26 to 222.70)	2 (98.61; 36.41, 72.87 to 124.36)	33.76 (−2.77 to 70.28); 0.069
Copper	86 (1537.47; 388.21, 733.44 to 2285.79)	4 (1856.58; 775.14, 1034.52 to 2884.45)	−319.11 (−733.32 to 95.10); 0.129	22 (1485.91; 373.43, 894.08 to 2255.59)	4 (1770.47, 310.24, 1441.90 to 2132.65)	−284.56 (−695.30 to 126.18); 0.166	39 (1568.10; 441.86, 733.44 to 2285.79)	2 (934.99; 206.40, 789.05 to 1080.84)	633.11 (−8.34 to 1274.55); 0.053
Iron	86 (1552.29; 515.24, 555.94 to 4354.18)	4 (1573.00; 272.05, 1321.53 to 1951.97)	−20.741 (−537.97 to 496.56); 0.937	22 (1515.34; 407.21, 818.46 to 2272.16)	4 (1489.00; 211.15, 1223.25 to 1728.39)	26.34 (−409.12 to 461.79); 0.902	39 (1460.26; 340.79, 925.26 to 2499.36)	2 (928.03; 526.21, 555.94 to 1300.12)	532.24 (23.68 to 1040.80); 0.041
Lead	2 (8.95; 4.31, 5.91–12.00)	0	--	1 (11.99; -, -)	0	--	0	0	--
Magnesium	86 (28.86; 5.45, 14.43 to 41.75)	4 (30.16; 3.49, 25.49 to 33.54)	−1.30 (−6.79 to 4.18); 0.638	22 (28.67; 5.18, 17.35 to 36.94	4 (33.51; 5.80, 29.20 to 41.75)	−4.83 (−10.74 to 1.07); 0.104	39 (28.12; 5.46, 14.43 to 41.75)	2 (18.76; 4.30, 15.72 to 21.80)	9.36 (1.39 to 17.32); 0.023
Manganese	9 (8.06; 3.60, 5.02–1489)	0	--	2 (10.24; 6.59, 5.58 to 14.89)	1 (5.10; -, -)	--	1 (5.94; -, -)	1 (9.9; -, -)	--
Mercury	8 (13.03; 14.75, 5.17–48.54)	0	--	1 (5.78; -, -)	0	--	3 (24.71; 20.81, 10.11 to 48.54)	0	--
Selenium	86 (122.70; 27.11, 52.44 to 188.95)	4 (134.15; 27.93, 93.94 to 157.95)	−11.45 (−39.04 to 16.14); 0.412	22 (117.61; 20.43, 66.34 to 146.58)	4 (142.32; 14.16, 126.19 to 159.86)	−34.72 (−46.88 to −2.55); 0.030	39 (119.34; 23.31, 58.80 to 159.86)	2 (73.95; 30.42, 52.44 to 95.45)	45.39 (10.90 to 79.89); 0.011
Zinc	86 (1160.13; 202.42, 603.60 to 1756.88)	4 (1183.55; 170.36, 1043.47 to 1431.52)	−23.42 (−228.16 to 181.31); 0.821	22 (1180.77; 171.57, 685.64 to 1431.52)	4 (1203.32; 112.63, 1043.47 to 1292.18)	−22.55 (−208.06 to 162.96); 0.804	39 (1142.39; 187.41, 685.64 to 1530.43)	2 (861.77; 365.11, 603.60 to 1119.94)	280.62 (−3.90 to 565.14); 0.053

## Data Availability

All data related to the current study, intended for reasonable use, is available from J.A.G. Agúndez (University Institute of Molecular Pathology Biomarkers, University of Extremadura -UNEx ARADyAL Instituto de Salud Carlos III, Av/de la Universidad S/N, E10071 Cáceres. Spain) and F.J. Jiménez-Jiménez (Section of Neurology, Hospital del Sureste, Arganda del Rey, Madrid, Spain).

## Supplementary Materials

The following supporting information can be downloaded at: https://www.mdpi.com/article/10.3390/antiox11020272/s1, Figure S1. Correlation between Calcium and Vitamin D levels in RLS patients; Figure S2. Correlation between Calcium and age at onset in RLS patients; Figure S3. Correlation between Magnesium and age at onset in RLS patients; Figure S4. Correlation between Manganese and IRLSSGRS in RLS patients; Table S1. Biological functions and effects of deficiency or toxicity of trace metals; Table S2. STROBE Statement.
