# Supplementary material for: Serum Trace Elements Concentrations in Patients with Restless Legs Syndrome
Source: Antioxidants (Basel). 2022 Jan 29;11(2):272. doi: 10.3390/antiox11020272 (PMC8868060; doi:10.3390/antiox11020272)
Supplement: Supplementary file 1 [file antioxidants-11-00272-s001.zip › antioxidants-1580242-supplementary.pdf]

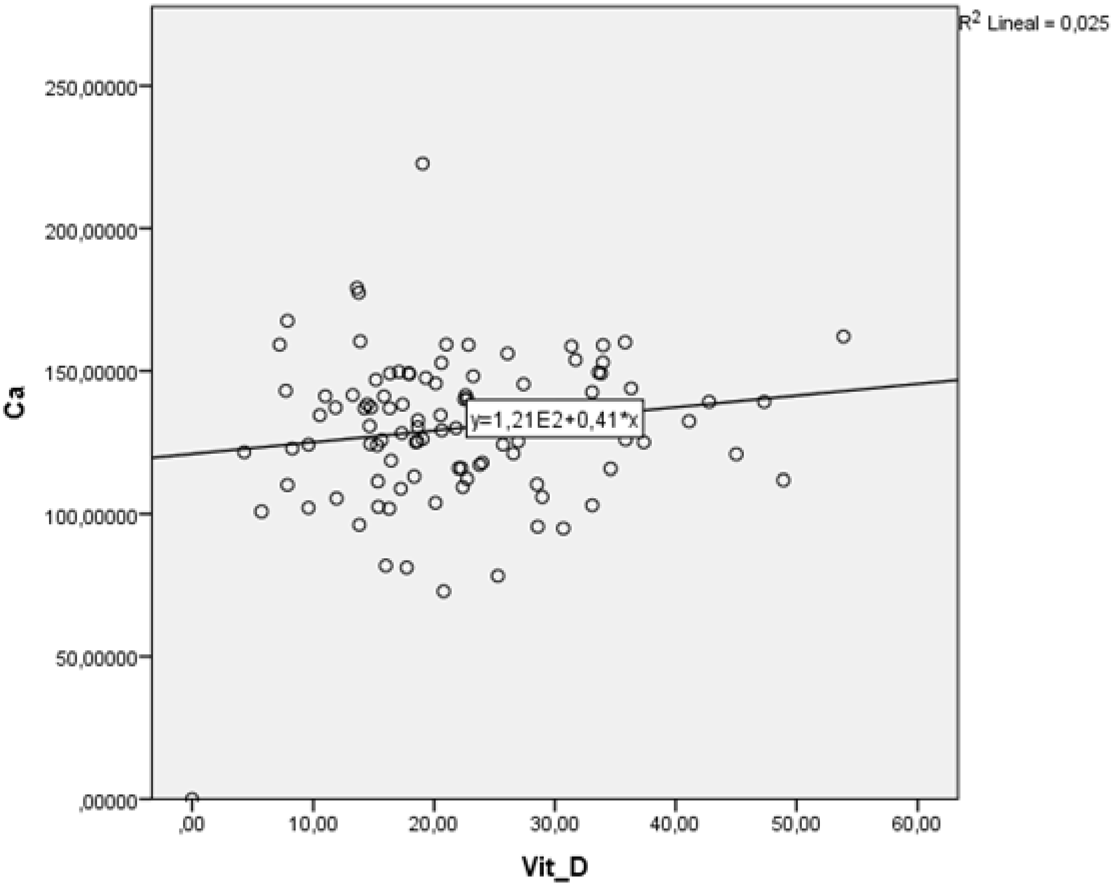

Figure S1. Correlation between Calcium and Vitamin D levels in RLS patients.

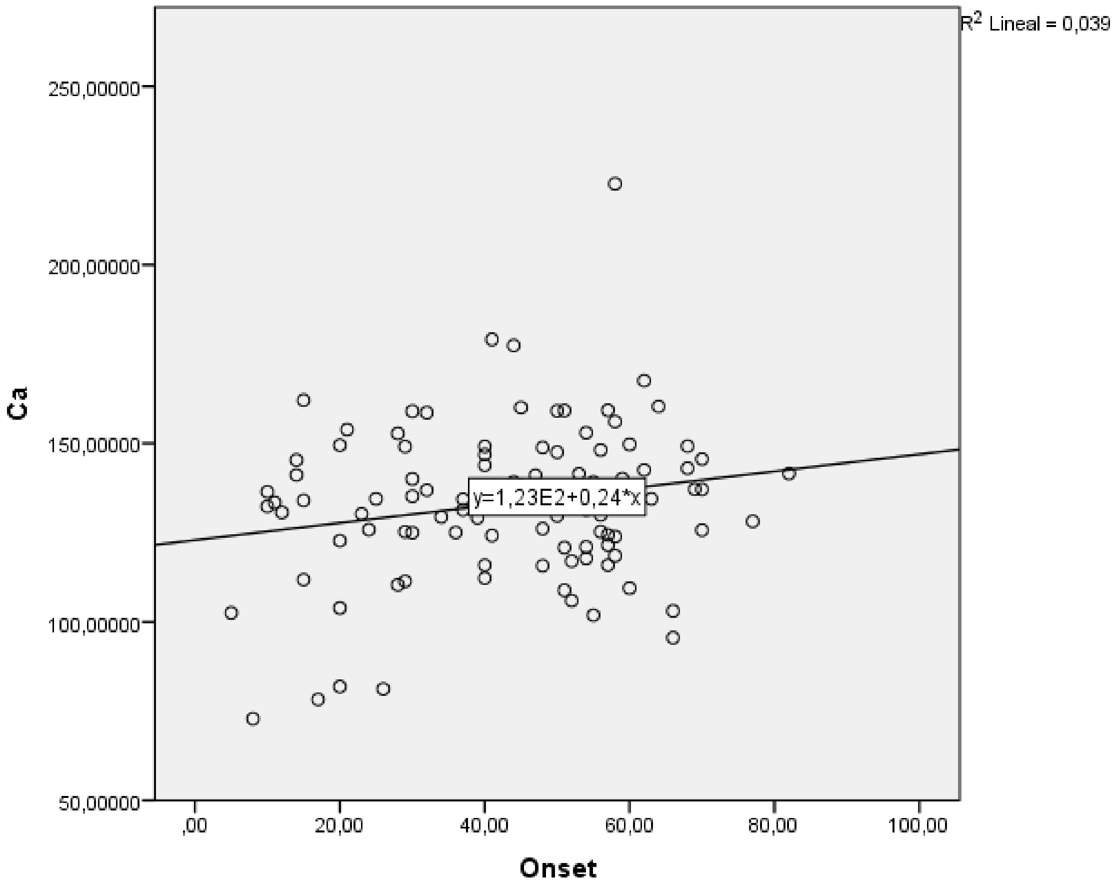

Figure S2. Correlation between Calcium and age at onset in RLS patients.

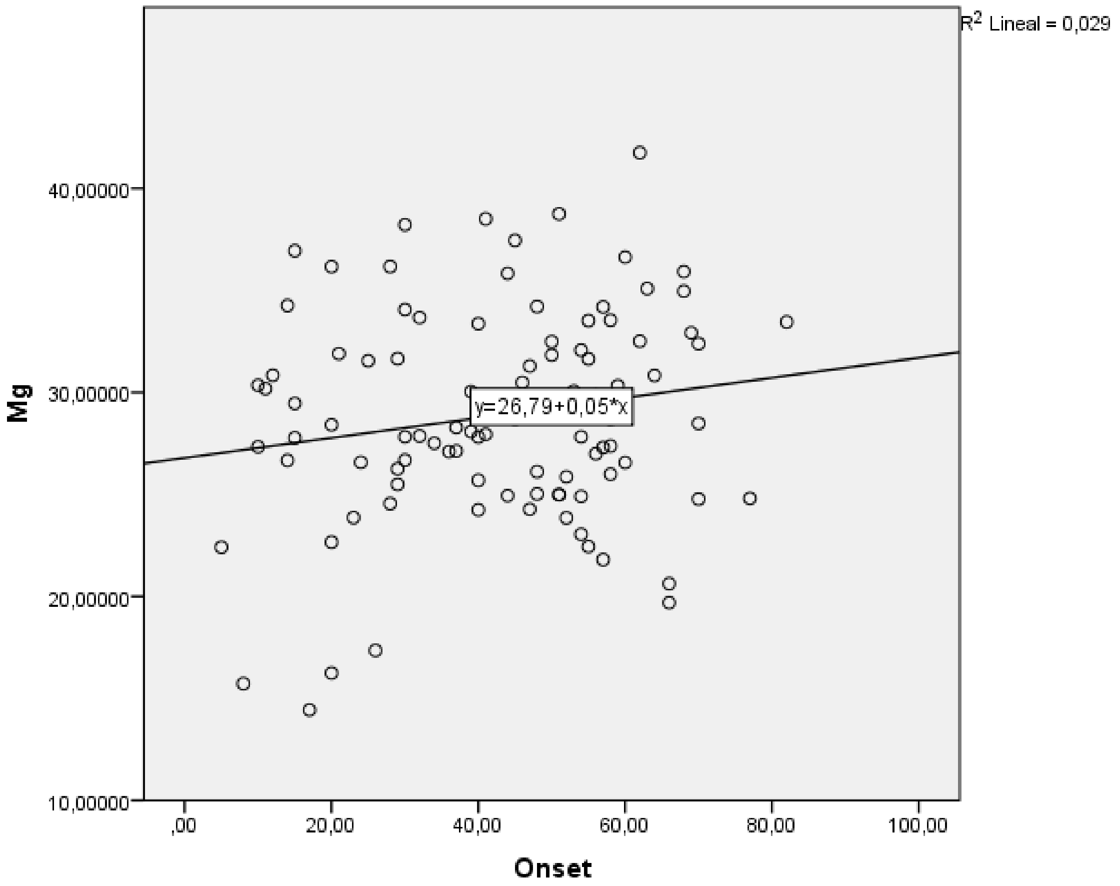

Figure S3. Correlation between Magnesium and age at onset in RLS patients.

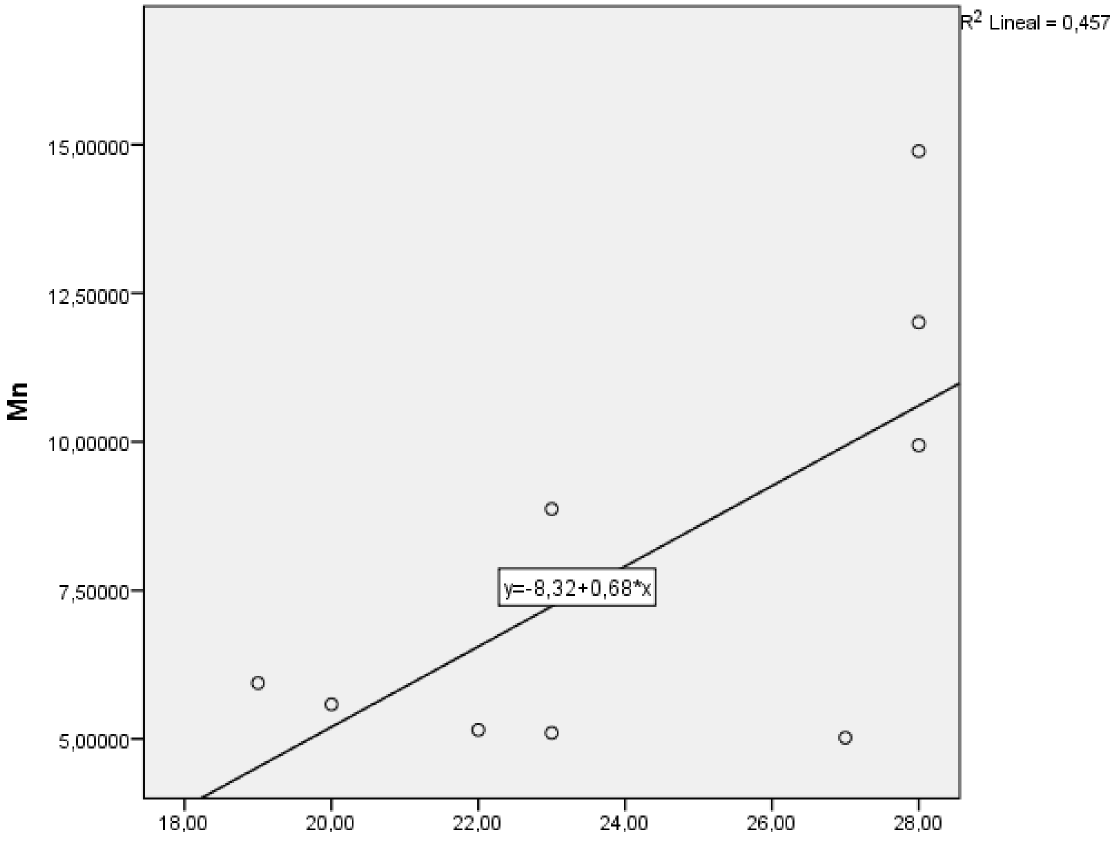

Figure S4. Correlation between Manganese and International Restless Legs Syndrome Study Group Rating Scale (IRLSSGRS) in RLS patients.

Table S1. Biological functions and effects of deficiency or toxicity of trace metals.

METAL [refs]	BIOLOGICAL FUNCTIONS	EFFECTS OF DEFICIENCY OR TOXICITY
Iron [9]	• The most abundant trace mineral, component of haeme, combines with globins to form haemoglobin in the bone marrow and participates in erythropoiesis.<br>• Involved in cell metabolism and regrowth of organisms.<br>• Present as iron-sulphur groups that contribute to physiological systems such as oxygen transport, deoxyribonucleic acid (DNA) synthesis, metabolic energy, cellular respiration, and electron transport in mitochondria.<br>• Partially stored as ferritin and haemosiderin in the liver, bone marrow, and muscle.<br>• Involved in the functioning of the immune system and cognition	• Iron deficiency can cause asthaenia (even without anaemia), reduction of immune defenses, aggravating immunosenescence leading to infections, impairment of brain function including cognition, worsening heart failure, hair loss, delayed skin healing, deafness, asymptomatic bacteriuria in overweight individuals, osteoporosis, restless legs syndrome, pruritus, and decreased physical activity.<br>• Iron overload can cause alterations in cognition, liver dysfunction (up to cirrhosis), cardiac consequences (heart failure and supraventricular or ventricular rhythm disturbances, cardiomyopathy), damage by accumulation in the spleen, bone marrow, pituitary gland, pancreas, and the central nervous system.
Copper [10, 11]	• Cofactor of numerous metalloproteins/metalloenzymes (Cu/Zn superoxide-dismutase, Lysil-oxidase, cytochrome c oxidase, dopamine beta hydroxylase, tyrosinase, and ceruloplasmin among others), intervening in cellular respiration, biosynthesis of neurotransmitters and peptide hormones, protection against free radicals, cross-linking of elastin, collagen, and keratin.<br>• Role in iron homeostasis, affecting indirectly haematopoiesis and participating in blood coagulation and angiogenesis.<br>• Role in nutritional immunity by acting as a component of the antimicrobial arsenal produced by cells of the innate immune system.<br>• Antibacterial, antifungal, and antiviral actions.<br>• Role in neurodegenerative processes in Alzheimer's, Parkinson's, Huntington's, and Creutzfeld-Jakob diseases.<br>• Relation with size, occurrence, progression, and recurrence of malignant tumours.<br>• Induction of proliferation and migration of endothelial cells by activating several angiogenic factors.	• Hypocupraemia can induce bone marrow dysplasia, myelopathy and a progressive ascending sensory and symmetric neuropathy associated with gait ataxia, cerebellar ataxia, fatigue, dyspnoea, macrocytic anaemia, neutropenia, and weight loss.<br>• Copper chronic toxicity causes liver and kidney damage, anaemia, immunotoxicity, developmental toxicity, and brain damage.<br>Indu
Manganese [12-15]	• Cofactor of numerous metalloproteins/metalloenzymes including Mn-superoxide dismutase (involved in defence against oxidative stress), phosphoenolpyruvate carboxykinase (involved in gluconeogenesis), pyruvate carboxylase (involved in gluconeogenesis and lipogenesis), acetyl-CoA decarboxylase (involved in fatty acid synthesis), arginase (involved in maintaining ammonia levels), glutamine synthetase (involved in the metabolism	• Accumulation preferably in the central nervous system, especially degeneration of dopaminergic neurons in the basal ganglia causing parkinsonian-like symptoms.<br>• Unsteady gait and muscular weakness.

	of glutamate to glutamine), glycosyltransferases (involved in bone mineralization), and protein serine/threonine phosphatase-1 (PP1, involved in cell survival and differentiation).<br>• Role in macronutrient metabolism.<br>• Role in the homeostasis of iron, copper, zinc, and calcium by competition in intestinal absorption.<br>• Role in insulin and insulin-growth cofactor signalling pathways.<br>• Effects on several neurotransmitter systems: dopaminergic (reduced dopamine levels by reduction of tyrosine hydroxylase activity, down-regulation of vesicular monoamine transporter 2 –VMAT2- and dopamine transporter -DAT-, and dopamine oxidation), GABAergic (increase in intracellular GABA levels mediated through inhibition of the GABA transporter GAT1), glutamatergic (reduction in glutamate uptake by astrocytes, by alteration of glutamate transporters, resulting in increased extracellular glutamate concentrations), and cholinergic (alteration in choline uptake, acetylcholine transferase activity, acetylcholine release and postsynaptic acetylcholine binding to clearance receptors).<br>• Role in intellectual development, neurobehaviour, and neuromotor functions.<br>• Role in neurodegenerative diseases including Parkinson's and Alzheimer's diseases.	• Learning deficits/cognitive problems.<br>• Neuropsychiatric symptoms (locura manganica)<br>• Inflammation in the lungs and respiratory symptoms<br>Indu
Zinc [16]	• Role in proper cellular functions, including differentiation, cell division, cell growth, cellular transport, protein synthesis, RNA and DNA synthesis, and DNA replication.<br>• Antioxidant actions, both directly preventing the formation of the reactive response of free radicals, or indirectly as a cofactor of Cu/Zn superoxide-dismutase, increasing glutathione peroxidase activity, and intervening in the synthesis of metallothioneins and metalloproteins/metalloenzymes.<br>• Cofactor for more than 1000 enzymatic reactions and more than 2000 transcription factors.<br>• Initiation and inhibition of apoptosis and regulation of inflammatory response.<br>• Role in learning and memory processes (it is present in the synaptic vesicles).<br>• Role in endocrine and immune system (both in cell-mediated and humoral immunity), and in bone formation.<br>• Inhibition of cancer cell proliferation and migration and promotion of apoptosis in cancer cells.<br>• Protection against toxicity of non-essential metals such as Pb, As, Hg, and Cd.	• Zinc deficiency may cause the affectation of immune and gastrointestinal systems, central nervous system, skeletal and reproductive systems.<br>• Zinc can induce neurotoxicity that participates in various brain disorders including stroke, traumatic brain injury, and seizures.
Selenium [17-19]	• Selenocysteine (recognized as a non-standard amino acid, it exists in the active site of many selenium-depending enzymes and in the structure of other selenoproteins), including glutathione-peroxidases and thioredoxin reductase, involved in oxidative stress.<br>• Activation and inactivation of thyroid hormone.<br>• Anti-inflammatory, antiviral and antioxidant activities.<br>• Participation in immune responses.	• Selenium deficiency is considered a potential risk for several types of cardiovascular disease (including Keshan disease), osteocondropathies with joint necrosis (Kashin-Beck disease), thyroid diseases, and certain myopathies. May cause affectation of immune and

	• Role in transcription, phospholipids biosynthesis, calcium flux in immune cells and endoplasmic reticulum-associated degradation, protein folding, muscle growth, and male reproduction. • Role in the foetal central nervous system development, in brain function maintenance, and in neurodegenerative diseases including Parkinson's and Alzheimer's diseases. • Possible role in the risk for diabetes mellitus, cardiovascular disease, and prevention of several types of cancer. • Role as a modulator in peripheral pain through inhibition of transient receptor potential (TRP) channels in the dorsal root ganglia.	gastrointestinal systems, central nervous system, skeletal and reproductive system. • Selenium toxicity (selenosis) is rare and includes hair and nail brittleness and loss, garlic-like breath odor, gastrointestinal disturbances, fatigue, and serious respiratory, renal, and cardiac complications.
Magnesium [20, 21]	• Mg2+ affects more than 600 enzymatic reactions, including energy metabolism, protein synthesis, and signal transduction. • Protection against oxidative stress processes. • Regulation of several ion channels, and modulation of N-methyl-D-aspartate (NMDA) glutamatergic receptor (important in developmental plasticity, learning and memory, and circadian clock rhythm). • DNA protection and genome stability (acting as a cofactor in the DNA-repair-mechanism-related enzyme and as a competitive inhibitor of the DNA-damaging factor due to the binding of Mg2+ to DNA). • Role in the stability of other biomolecules such as RNA and proteins. • Role in growth and differentiation at cellular and tissue levels.	• Magnesium toxicity is more frequent in patients with chronic kidney disease under haemodialysis, patients undergoing cancer treatment, and women receiving preeclampsia treatment. • Acute toxicity includes hypotension, nausea, vomiting, facial flushing, urine retention, ileus, depression, lethargy, muscle weakness, and difficulty breathing. • Disorder of Mg2+ homeostasis is involved in cancer, diabetes, and neurodegenerative diseases, such as Parkinson's disease (PD), Alzheimer's disease (AD), and demyelination.
Calcium [22, 23]	• Control of nerve excitability. • Maintenance of the integrity of the skeletal muscles. • Maintenance of the tone and contractility of the heart. • Formation of bones, teeth, and other tissues. • Role in blood clotting and blood pressure regulation. • Regulation of hormone function. • Actions as second messenger and enzyme cofactor	• Calcium deficiency induces muscle cramping (tetany), dry skin and brittle nails, bone fractures, seizures, arrhythmias, and cardiac block. • Chronic form of hypercalcemia can cause neurological and neuromuscular disturbances such as adynamia, vomiting, gastric ulcers, pancreatitis, renal failure, and cardiac arrhythmia (including cardiac arrest) or hypertension
Aluminium [24]	• No known biological function in humans	• Alterations in bioavailability of essential elements such as calcium, iron, zinc, and copper. • Toxicity includes osteomalacia, microcytic anaemia, and adverse neurological effects (muscular contractions, ataxia, difficulties in speech and in swallowing, convulsions and dementia)

Lead [11]	• No known biological function in humans	• Anaemia, gingival (Burton's line), and dental changes.<br>• Brain toxicity (lead encephalopathy)<br>• Induction of a progressive, asymmetric motor polyneuropathy, with a predominant affectation of upper limbs.
Cadmium [15]	• No known biological function in humans	• Defects in liver, bones, kidneys, lungs, testes, brain, immunological and cardiovascular systems.<br>• Induction of axonal sensorimotor neuropathy
Arsenic [11, 15, 24]	• No known biological function in humans, although probably plays a physiological role in methionine metabolism,	• Defects in liver, bones, kidneys, lungs, testes, brain, immunological and cardiovascular systems<br>• Induction of subacute progressive sensory-motor polyneuropathy with autonomic dysfunction.
Mercury [15, 24]	• No known biological function in humans	• Accumulation preferably in the central nervous system, causing impairment in motor coordination, disturbances in learning and memory processing, depressive-like behaviour, parkinsonian-like syndrome.<br>• Induction of axonal, sensory polyneuropathy with small-fibre and autonomic dysfunction.<br>• Toxic effects on the kidney (nephrotic syndrome) cardiovascular, endocrine, and immune system.

Table S2. STROBE Statement. Checklist of items that should be included in reports of observational studies.

Section/Topic	Item No	Recommendation	Reported on Page No
**Title and abstract**	1	(a) Indicate the study's design with a commonly used term in the title or the abstract	2
		(b) Provide in the abstract an informative and balanced summary of what was done and what was found	2
		**Introduction**	
Background/rationale	2	Explain the scientific background and rationale for the investigation being reported	3,4, Supplementary Table 1
Objectives	3	State specific objectives, including any prespecified hypotheses	4
		**Methods**	
Study design	4	Present key elements of study design early in the paper	4,5
Setting	5	Describe the setting, locations, and relevant dates, including periods of recruitment, exposure, follow-up, and data collection	4,5
Participants	6	(a) Cohort study—Give the eligibility criteria, and the sources and methods of selection of participants. Describe methods of follow-up	4-6

		Case-control study—Give the eligibility criteria, and the sources and methods of case ascertainment and control selection. Give the rationale for the choice of cases and controls<br>Cross-sectional study—Give the eligibility criteria, and the sources and methods of selection of participants	
		(b) Cohort study—For matched studies, give matching criteria and number of exposed and unexposed<br>Case-control study—For matched studies, give matching criteria and the number of controls per case	4-6
Variables	7	Clearly define all outcomes, exposures, predictors, potential confounders, and effect modifiers. Give diagnostic criteria, if applicable	4-6
Data sources/measurement	8*	For each variable of interest, give sources of data and details of methods of assessment (measurement). Describe comparability of assessment methods if there is more than one group	4-7
Bias	9	Describe any efforts to address potential sources of bias	6-7
Study size	10	Explain how the study size was arrived at	6-7
Quantitative variables	11	Explain how quantitative variables were handled in the analyses. If applicable, describe which groupings were chosen and why	6-7
Statistical methods	12	(a) Describe all statistical methods, including those used to control for confounding	5-6
		(b) Describe any methods used to examine subgroups and interactions	5-7
		(c) Explain how missing data were addressed	NA
		(d) Cohort study—If applicable, explain how loss to follow-up was addressed<br>Case-control study—If applicable, explain how matching of cases and controls was addressed<br>Cross-sectional study—If applicable, describe analytical methods taking account of sampling strategy	5-7
		(e) Describe any sensitivity analyses	5-7
**Results**			
Participants	13*	(a) Report numbers of individuals at each stage of study—eg numbers potentially eligible, examined for eligibility, confirmed eligible, included in the study, completing follow-up, and analysed	6,7
		(b) Give reasons for non-participation at each stage	NA
		(c) Consider use of a flow diagram	NA
Descriptive data	14*	(a) Give characteristics of study participants (eg demographic, clinical, social) and information on exposures and potential confounders	6,7
		(b) Indicate number of participants with missing data for each variable of interest	NA
		(c) Cohort study—Summarise follow-up time (eg, average and total amount)	NA
Outcome data	15*	Cohort study—Report numbers of outcome events or summary measures over time	NA
		Case-control study—Report numbers in each exposure category, or summary measures of exposure	6-7<br>TABLES 1, 3 and 4, Suppleme

			ntary Figures 1 to 4
		Cross-sectional study—Report numbers of outcome events or summary measures	NA
Main results	16	(a) Give unadjusted estimates and, if applicable, confounder-adjusted estimates and their precision (eg, 95% confidence interval). Make clear which confounders were adjusted for and why they were included	6-7<br>TABLES 1, 3 and 4, Suppleme ntary Figures 1 to 4
		(b) Report category boundaries when continuous variables were categorized	NA
		(c) If relevant, consider translating estimates of relative risk into absolute risk for a meaningful time period	NA
Other analyses	17	Report other analyses done—eg analyses of subgroups and interactions, and sensitivity analyses	6-7<br>TABLES 1, 3 and 4, Suppleme ntary Figures 1 to 4
**Discussion**			
Key results	18	Summarise key results with reference to study objectives	7-9
Limitations	19	Discuss limitations of the study, taking into account sources of potential bias or imprecision. Discuss both direction and magnitude of any potential bias	9
Interpretation	20	Give a cautious overall interpretation of results considering objectives, limitations, multiplicity of analyses, results from similar studies, and other relevant evidence	9
Generalisability	21	Discuss the generalisability (external validity) of the study results	9
**Other Information**			
Funding	22	Give the source of funding and the role of the funders for the present study and, if applicable, for the original study on which the present article is based	9
